# Upper arm movements in the last days of life: A new possible sign of impending death

**DOI:** 10.1017/S1478951524002001

**Published:** 2025-01-23

**Authors:** Miguel Julião, Carolina Simões, Patrícia Calaveiras, Paula Câmara, Miguel Castelo-Branco

**Affiliations:** 1 Department of Palliative Medicine, Equipa Comunitária de Suporte em Cuidados Paliativos, ULS Amadora/Sintra, Sintra, Portugal; 2 Faculdade de Medicina da Universidade de Coimbra, Universidade de Coimbra, Coimbra, Portugal

**Keywords:** Palliative care, signs of impending death, upper arm movements, home-based palliative care, terminally ill

## Abstract

One of the most crucial stages of palliative care is the last days and hours of life, which require special attention and knowledgeable identification of clinical signs described as signs of impending death (SID). Our case series of 11 patients receiving home palliative care describes bilateral hypoactive, stereotyped upper arm movements (scratching of the head, forehead, and nose) that were previously unknown or described, often accompanied by SID.

## Introduction

Palliative care is an approach that aims to enhance the quality of life of patients and their families who are dealing with life-threatening illnesses. Palliative care is crucial and should be initiated as early as possible in the disease’s course. The support provided does not end with the patient’s death but continues until the family completes the grieving process.

One of the most crucial stages of palliative care is the last days and hours of life, which require special attention. It is essential to identify this stage to make appropriate medical decisions, discontinue potentially futile interventions, and communicate effectively with relatives and friends. Significant challenges in this context include preparing the family for what they can expect during this period and what is considered the best care.

Some clinical signs are already described as signs of impending death (SID) that help us identify patients who are in their last days or hours of life. These include dysphagia, a performance status of 20 or less, death rattle, decreased level of consciousness, Cheyne–Stokes respiration, livedo reticularis/peripheral cyanosis, decreased or absent urinary output, drooping of the nasolabial fold, fetor hepaticus, and delirium (Ellershaw J & Ward C, 2003; Hui D, et al., 2014; 2015a; 2015b; Morita T, et al., 1998; Plonk WM, Jr. & Arnold RM, 2005, Sobral et al., 2019; Ventafridda V, et al., 1990). The presence of SID can be distressing for relatives. Many of them may find that their loved one is exhibiting SID as a sign of suffering (Hui D, et al., 2014; 2015a; 2015b). Therefore, open communication and providing a simple and adapted pathophysiological explanation of SID are crucial. It is important to listen to how the relatives make sense of their observations of the signs to ease their suffering and allow them to invest in other areas of being with their loved one during their last hours or days of life without any misconceptions.

**Figure 1.**
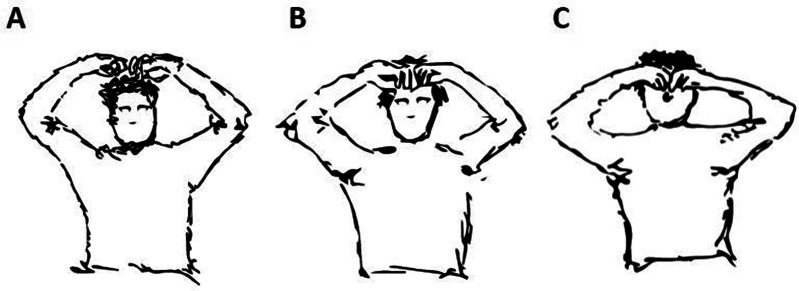
Drawings of upper arm movements. (A) upper arms on top of the head; (B) upper arms on forehead; and (C) upper arms on nose. Copyright: Julião M.

During regular medical visits, our team noticed bilateral hypoactive, stereotyped upper arm movements (UAM) that were previously unknown or described. Additionally, some UAM were referred by patients’ relatives or caregivers, in addition to the team’s observations. These movements included scratching of the head, forehead, and nose (Fig. 1, for drawings). The movements were often accompanied by SID, which led the team to question whether these movements were a part of SID or not. This case series aims to describe these 3 newly observed UAM in 11 patients receiving home palliative care and hypothesize their underlying neurological factors.

## Methods

This is a retrospective analysis of all patients who were cared for by our team between 2022 and 2024 and who had experienced at least one of the newly observed movements, whether accompanied by any other SID or not. We have collected the following data: (1) demographic and clinical characteristics of the patients; (2) presence of SID; (3) types of movement(s) observed; (4) presence of agitation and delirium based on clinical evaluation; (5) time to death since the appearance of movements; and (6) feedback from family members or caregivers regarding the observed movement and their feelings toward it. Our study did not require approval from an institutional review board because it involved analyzing anonymous existing data that did not directly impact patients or caregivers.

## Results

Eleven cases were reviewed, which involved 11 individuals with advanced diseases. Seven of them were male, and their average age was 75 years, ranging from 58 to 99 years. Ten patients had cancer, 5 of which had metastasized to advanced stages, and 2 had dementia. Only 1 patient did not have any accompanying SID (patient #8, Table 1). The palliative performance status mean was 15%, ranging from 10% to 30%. Of the 11 observed patients, 6 had a history of smoking and 5 had history of other risk factors for cardiovascular diseases. Table 1 summarizes the case series, including patients’ characteristics and caregivers’ feedback. After speaking with relatives and caregivers, it was discovered that the movements appeared to be purposeless, without any precipitant factor and lasted between 5 and 60 minutes, repeated several times a day (numbers referred by caregivers based on memory recall). Another observation was that when caregivers attempted to stop the movement by forcing the upper arms back toward the torso, patients’ movements toward their heads would slowly return and persist, with no other abnormal muscular patterns like myoclonus, chorea, and dystonia.

**Table 1.** Case series characteristics

Pt	Age	Gender	Diagnosis & clinical condition	Scratching head movement	Scratching forehead movement	Scratching nose movement	Agitation	Time to death since the appearance of movements, *days*	Family/caregivers noticing and feelings about movements
1	83	M	- Colon cancer metastasized to liver and lungs	Present bilaterally^a^	Present bilaterally	Present bilaterally	None	5	- Family notices and alerts team
			-Ex-smoker						
			- Dependency in ADL						
			- Bed bound						
			- PPS^b^: 20%						- Strangeness
			- SID:						
			Dysphagia						- Slight anxiety
			Superficial breathing pattern						
			Cervical hyperextension						
2	89	F	- Advanced-stage dementia	Present bilaterally	Present bilaterally	None	None	5	- Strangeness
			- Terminal heart failure						
			- Hypertension						
			- Type 2 DM						
			- Last days of life						
			- Dependency in ADL						
			- Bed bound						
			- PPS: 20%						
			- SID:						
			Dysphagia						
			Superficial breathing pattern						
			Peripheral cyanosis						
3	74	M	- Stage IV kidney cancer	None	Present bilaterally	None	None	3	-
			- Hypertension						
			- Ex-smoker (20 pack-year)						
			- Benign prostatic hyperplasia						
			- Last days of life						
			- Dependency in ADL						
			- Bed bound						
			- PPS: 10%						
			- SID:						
			Dysphagia						
			Superficial breathing pattern						
			Peripheral cyanosis						
4	69	F	- Stage III ovarian cancer metastasized to liver and peritoneum	Present bilaterally	Present bilaterally	None	None	2	-
			- Last days of life						
			- Dependency in ADL						
			- Bed bound						
			- PPS: 20%						
			- SID:						
			Dysphagia						
			Hypoactive delirium						
			Peripheral cyanosis						
5	65	F	- Advanced tongue cancer	Present bilaterally	None	Present bilaterally	None	2	- Slight anxiety
			- Ex-smoker (40 pack-year)						
			- Last days of life						
			- PPS: 10%						
			- SID:						
			Cervical hyperextension						
			Drooping of nasolabial fold						
			Cheyne–Stokes respiration						
6	58	F	- Pancreatic cancer	None	Present bilaterally	None	None	1	-
			- Hypertension						
			- Dyslipidemia						
			- Last hours of life						
			- PPS: 10%						
			- SID:						
			Cervical hyperextension						
			Dysphagia						
			Livedo reticularis/peripheral cyanosis						
			Drooping of nasolabial fold						
7	87	M	- Pancreatic cancer	None	Present bilaterally	Present bilaterally	Mild agitation	1	- Strangeness
			- Bladder cancer						
			- Hypertension						
			- Dyslipidemia						
			- Type 2 DM						
			- Ex-smoker (40 pack-year)						
			- Last hours of life						
			- PPS: 10%						
			- SID:						
			Oromandibular respiration						
			Dysphagia						
			Death rattle						
			Livedo reticularis/peripheral cyanosis						
			Drooping of nasolabial fold						
8	60	M	- Stage IV melanoma	Present bilaterally	Present bilaterally	None	- Mild agitation	11	-
			- Depression						
			- Ex-smoker				- Mild agitation		
			- PPS: 30%						
9	66	M	- Lung cancer	Present bilaterally	Present bilaterally	None	None	1	-
			- Larynx cancer						
			- Ex-smoker (40 pack-year)						
			- Last hours of life						
			- PPS: 10%						
			- SID:						
			Oromandibular respiration						
			Dysphagia						
			Cheyne–Stokes respiration						
			Livedo reticularis/peripheral cyanosis						
			Fetor hepaticus						
10	74	M	- Pancreatic cancer metastasized to liver	None	None	Present bilaterally	None	1	- Family notices and alerts team
			- Type 2 DM						
			- Dyslipidemia						
			- Ex-smoker (40 pack-year)						
			- Last hours of life						
			- PPS: 10%						
			- SID:						
									- Strangeness
			Oromandibular respiration						
			Dysphagia						
			Cheyne–Stokes respiration						- Slight anxiety
			Livedo reticularis/peripheral cyanosis						
			Drooping of nasolabial fold						
			Fetor hepaticus						
11	99	M	- Slight dementia	Present bilaterally	Present bilaterally	None	None	1	- Strangeness
			- Geriatric frailty						
			- Pneumonia						
			- Last hours of life						
			- PPS: 10%						
			- SID:						
			Oromandibular respiration						- Slight anxiety
			Dysphagia						
			Cheyne–Stokes respiration						
			Livedo reticularis/peripheral cyanosis						
			Drooping of nasolabial fold						
			Cervical hyper extension						

ADL, activities of daily life; DM, diabetes mellitus; SID, signs of impending death; PPS, Palliative Performance Scale; Pt, patient.

a Present bilaterally means with both arms elevated. ^b^PPS: 100% – totally healthy; 0% – death.

## Discussion

The agonic phase, a physiological state preceding the final days or hours of terminally ill patients, often manifests with multiple SID, reflecting multi-organ failure in various vital systems including the brain, heart, lungs, and kidneys. Recognizing SID at the patient’s bedside is pivotal in palliative care, influencing not only patients and their families but also health-care providers, impacting communication and end-of-life decisions. Despite its significance, there remains limited understanding of identifying SID in the final stages of life (Hui et al., 2014).

To the best of our knowledge, our case series marks a pioneering effort in documenting new UAM occurring during the terminal phase of 11 persons cared for by our home-based palliative care team. We sought to discern if these movements signify a distinct type of SID or are exclusive to the studied patients. Additionally, we also wanted to speculate on the possible neurological pathways involved in such observed movements. The observed movements are quite distinct from other abnormal patterns observed in neurocritical patients such as chorea, dystonia, and myoclonus (Hannawi Y, et al., 2016; Jain S & DeGeorgia M, 2005). These and other manifestations have often a common cause in basal ganglia neurovascular integrity, and we therefore hypothesize that the basal ganglia may underlie these movement patterns, possibly due to failure of the physiological pallidal inhibition that may be disrupted in these patients (Demirayak P, et al., 2018). A cerebellar origin is less likely, because these would more often lead to oscillatory or tremor-like patterns. Hemiballistic patterns would be more consistent with subthalamic dysfunction.

A finding of high significance is the mirror movement pattern. This is reminiscent of congenital mirror movement disorder (CMMD) which is characterized by unintended, nonsuppressible, homologous mirroring activity (Demirayak et al., 2018). Other causes of this type of movement include as Klippel–Feil syndrome, X-linked Kallman syndrome, ischemic stroke, and hemiplegic cerebral palsy. A recent functional magnetic resonance imaging study showed that mirror movements in CMMD are highly correlated with abnormal neuronal activity not only in cortex but also in subcortical structures, involving the basal ganglia (Demirayak et al., 2018). In sum, this pattern may emerge from disrupted cortico-striatal-thalamic-cortical loops leading to abnormal patterns of symmetric activity in the supplementary motor area and primary motor cortex, even when unimanual movements are planned. The findings observed here may involve the same cortical-subcortical pathways.

To date, there is no existing published literature on these newly observed movements, besides a vague reference in an online reference.

Our report has some significant findings, some of which seem to support the hypothesis that UAM might be part of the constellation of SID.

First, our findings suggest a plausible link between UAM and SID, supported by the majority of patients (10 of 11) displaying UAM alongside other SID. Moreover, all patients died within the typical timeframe of the agonic phase after UAM onset. It is possible to classify the 1 patient who exhibited UAM and mild agitation, along with visual hallucinations before death, as potentially having SID, even if only these 2 neuro-cognitive possible SID were present in the last days of life. It is also possible that this patient’s UAM could be part of an isolated hypoactive delirium episode.

Consistently, UAM observed were predominantly hypoactive and bilateral, often occurring in clusters within the same individual. Notably, these movements appeared spontaneous, devoid of external stimuli, indicating a possible intrinsic physiopathological phenomenon, which we hypothesize being related with the basal ganglia. Moreover, our study unveiled associations between UAM and cardiovascular risk factors like hypertension, diabetes, and smoking, underscoring potential avenues for deeper exploration.

While acknowledging the limitation of our small sample size, it is worth noting that most cases of UAM were observed in male cancer patients. However, this may be due to our team’s referral patterns, and further investigation is needed to determine the prevalence of UAM across different patient demographics (as most UAM were observed in male patients) and diseases. It is important to examine if UAM exhibits different patterns depending on the specific disease progression, such as in advanced organ failures like chronic obstructive pulmonary disease or advanced heart failure. Furthermore, the number of pediatric referrals in our team is minimal, and future research should consider collaborating with pediatric centres to determine if such UAM are also present in this population.

As the team was alerted by caregivers and observed these particular types of UAM only, it might be biased to focus only on these 3 and neglect other possible UAM that may have similar underlying factors. In future clinical observations, other possible neurological-type SID cases should be observed, registered and described in close collaboration with families and caregivers, using the present UAM ones as a basis.

One of the significant findings in our report that requires attention is related to the observation of several caregivers and terminally ill individuals who were impressed and distressed by UAM. These movements were not only observed, but caregivers also tried to stop them, thinking they could indicate patients’ discomfort or special needs during their end-of-life stage. Caregivers’ feedback was essential to the team’s awareness and registration, providing more detailed information on the observed movements. By exploring the impact of UAM, health-care professionals can support patients and families better, who may experience fear and anxiety while observing these movements. Providing clear scientific hypotheses on UAM and explaining it to caregivers can alleviate their concerns, similar to how family conferences can prepare them for other SID present in end of life. In the future, in order to understand and study the different types of movements in more detail, it will be crucial to collaborate with caregivers and obtain their informed consent to identify and register movements using video or photographs. It is important to note that currently, the team does not have any photographic or video records available for analysis.

Our study has some limitations, some of which were already pointed earlier in the text. We conducted a retrospective analysis based on the existing information written in clinical processes. It is possible that more patients experienced these movements, but they were not registered. It is crucial to train the team of health professionals to increase awareness of UAM and the need to register it using written registries, photographs, or videos after obtaining informed consent from patients and caregivers. This will help improve the accuracy and completeness of future data.

We acknowledge that our sample size is limited. Therefore, while presenting 11 cases, we remain mindful that our findings cannot be generalized. Further research is required, using larger sample sizes and multicentric methodology. Although the frequency of UAM is lower compared to other frequent SIDs, we cannot draw any conclusions on its prevalence, mainly due to unregistered or unobserved cases during clinical activity.

We are also aware that the majority of our sample were patients with cancer, and this might be biased by the referral pattern of palliative care worldwide still and to our team equal. Deeper understanding of the UAM phenomenon should rely on research using other populations and diseases, where collaborations with pediatric centers are crucial.

It should be noted that most of the people we observed had cancer, which could have influenced our findings. To gain a better understanding of the UAM phenomenon, it would be beneficial to conduct research on other populations and diseases. Collaboration with pediatric centers is crucial to improve our team’s low percentage of pediatric palliative care follow-ups in UAM research.

Our research provides insights into the complex manifestations of SID during the agonic phase. It highlights the need for broader research to understand the progression of terminal illness across various patient populations and disease spectrums. UAM is believed to be a part of SID, and our study can enhance our understanding of the agonic phase, helping us identify patients in their last days or hours of life. Future research should focus on noninvasive techniques to uncover the specific underlying pathophysiological factors that contribute to UAM expression. Advancements in SID and UAM knowledge can provide more information on best practices for identifying SID in the last days or hours of life. This knowledge could lead to specific training for health-care professionals, as well as guidelines for communicating with patients and families during this critical time. This could ultimately facilitate a more peaceful and tranquil end-of-life phase for those who are losing and grieving their loved ones.
